# Remarks on the Seasonal Incidence of Diarrhœa, and the Part Played by Flies

**Published:** 1903-02-07

**Authors:** J. T. C. Nash


### HOSPITAL CLINICS AND MEDICAL PROGRESS.

### REMARKS ON THE SEASONAL INCIDENCE OF DIARRHŒA, AND THE PART PLAYED BY FLIES.

By J. T. C. NASH, M.D.EDIN., D.P.H.CANTAB.

*(Continued from p. 299.)*

Some samples of milk are no doubt contaminated by the use of unboiled contaminated water for washing the utensils as in the Victoria Park, Manchester, 1894 epidemic; this was an instance of direct infection of milk by human agency. The point, however, that I wish to impress is that I believe that flies are the chief agents concerned in carrying fœcal pollution to milk during the summer months, and are *facile princeps* as the chief indirect causal agents in summer diarrhœa, just as mosquitos are the chief indirect causal agents of intermittent or malarial fever.

My experience of epidemic diarrhœa at Southend during 1902 has given me some ground for advancing this hypothesis. During July and August there was no mortality among infants under one year from diarrhœa,[1] as compared with 23 deaths from the same cause during the same period of 1901. July and August 1902 were comparatively cool months and rain fell on 22 out of the 62 days (there being 13 wet days in August). The most remarkable phenomenon of these months was, however, the almost complete absence of musca domestica. In September this insect made its appearance, and, coincidentally, epidemic diarrhœa made its appearance and 13 infantile deaths[1] from this cause were recorded in three weeks. Unfortunately there was no 4 feet earth thermometer in Southend at the time, but I learn from Dr. Newman, Medical Officer of Health for Finsbury, that the 4 feet earth thermometer had attained to over 56° Fahr. several weeks before that date. The advent of the fly was of short duration, and the insect had practically disappeared again by September 25th. From this date no further deaths from diarrhœa were recorded in Southend until November, when a solitary death from enteritis appeared in the death returns.

Where refuse and midden heaps are in the proximity of cowsheds and dairies, they are, I am sure, a source of great danger even if the water supply is pure and beyond suspicion. I think they are a danger, partly, and to a lesser degree by the risk of infective dust being blown on to exposed milk during dry weather—and partly, and to a greater degree—by the risk of flies carrying contamination direct from the refuse heap to the milk, or to the udders of the cow, or the hands of the milker. Subsequent to this method of infection, time and temperature (as shown by Professor Delépine) play an important part in the increase of infective material, not to speak of fresh contamination by flies and dust in the dairy shops and in the homes of consumers.

In addition to the all-important preventive measures which should be procured at the various cowsheds and dairies as formulated in Professor Delépine's paper, I would add the covering over of all standing milk so as to absolutely prevent the access of flies. How often one sees a large uncovered bowl of milk on a counter labelled "Pure Milk" exposed to the dust, and with perhaps one or two flies floating in it, and several others skirting its edges. "Pure Milk," indeed! "Bacteria-breeding Poison" would be more appropriate. In the house it is still essential to insist on the observance of all measures which conduce to cleanliness and the protection of milk, etc., from contamination. In June 1902, I published in Southend-on-Sea a pamphlet containing certain rules and precautions to be observed by mothers of households. Some of these have been insisted upon previously by various sanatarians. The rules are, I think, so important (though so obvious and simple) that I make no apology for recording them in a paper read before so learned a society as this. I have just added to these rules certain additional precautions on the importance of excluding flies from milk, etc. The taste of boiled milk is very generally objected to. The method of meeting this objection, by heating the milk in a saucepan placed in a larger saucepan of water which is then made to boil for 20 minutes, is very useful and perfectly satisfactory. The milk is thus heated up to over 200° F., but does not itself boil. In connection with this matter a recent discovery by Dr. Gärtner[2] is of considerable value and importance. He finds that the peculiar taste of boiled milk depends upon contact with the air, and that it can be prevented by the addition of a small piece of paraffin wax to the heated milk. A surface air-tight layer of paraffin forms on the milk which can be removed afterwards. Paraffin is insoluble in milk and without odour, its action is therefore purely mechanical and unobjectionable.

[Appended to the above article are some "Simple rules for preventing many infantile complaints" which have been drawn up by Dr. Nash, who is Medical Officer of Health for the Borough of Southend-on-Sea, and printed by order of the Health Committee. In these rules stress is laid on the necessity of keeping all food, and especially milk, protected from flies.]

[1] Deaths from enteritis are included.
[2] Quoted in the "British Food Journal," Dec. 1902.

